# Blind bedside postpyloric placement of spiral tube as rescue therapy in critically ill patients: a prospective, tricentric, observational study

**DOI:** 10.1186/s13054-017-1839-2

**Published:** 2017-09-26

**Authors:** Bo Lv, Linhui Hu, Lifang Chen, Bei Hu, Yanlin Zhang, Heng Ye, Cheng Sun, Xiunong Zhang, Huilan Lan, Chunbo Chen

**Affiliations:** 1 Department of Critical Care Medicine, Guangdong General Hospital, Guangdong Academy of Medical Sciences, 106 Zhongshan Er Road, Guangzhou, 510080 Guangdong Province People’s Republic of China; 2 School of Medicine, South China University of Technology, Guangzhou Higher Education Mega Center, Guangzhou, 510006 Guangdong Province People’s Republic of China; 3 Department of Critical Care Medicine, Xinjiang Kashgar Region’s First People’s Hospital, 66 Airport Road, Kashgar Region, 844099 Xinjiang Uygur Autonomous Region People’s Republic of China; 4 Department of Critical Care Medicine, Guangzhou Nansha Central Hospital, 105 Fengzhedong Road, Guangzhou, 511457 Guangdong Province People’s Republic of China

**Keywords:** Blind bedside, Postpyloric placement, Spiral nasojejunal tube, Rescue therapy, Enteral nutrition, Critically ill patients

## Abstract

**Background:**

Various special techniques for blind bedside transpyloric tube placement have been introduced into clinical practice. However, transpyloric spiral tube placement facilitated by a blind bedside method has not yet been reported. The objective of this prospective study was to evaluate the safety and efficiency of blind bedside postpyloric placement of a spiral tube as a rescue therapy subsequent to failed spontaneous transpyloric migration in critically ill patients.

**Methods:**

This prospective, tricentric, observational study was conducted in the intensive care units (ICUs) of three tertiary hospitals. A total of 127 consecutive patients with failed spontaneous transpyloric spiral tube migration despite using prokinetic agents and still required enteral nutrition for more than 3 days were included. The spiral tube was inserted postpylorically using the blind bedside technique. All patients received metoclopramide intravenously prior to tube insertion. The exact tube tip position was determined by radiography. The primary efficacy endpoint was the success rate of postpyloric spiral tube placement. Secondary efficacy endpoints were success rate of a spiral tube placed in the third portion of the duodenum (D3) or beyond, success rate of placement in the proximal jejunum, time to insertion, length of insertion, and number of attempts. Safety endpoints were metoclopramide-related and major adverse tube-associated events.

**Results:**

In 81.9% of patients, the spiral feeding tubes were placed postpylorically; of these, 55.1% were placed in D3 or beyond and 33.9% were placed in the proximal jejunum, with a median time to insertion of 14 min and an average number of attempts of 1.4. The mean length of insertion was 95.6 cm. The adverse event incidence was 26.0%, and no serious adverse event was observed.

**Conclusions:**

Blind bedside postpyloric placement of a spiral tube, as a rescue therapy subsequent to failed spontaneous transpyloric migration in critically ill patients, is safe and effective. This technique may facilitate the early initiation of postpyloric feeding in the ICU.

**Trial registration:**

Chinese Clinical Trial Registry, ChiCTR-OPN-16008206. Registered on 1 April 2016.

## Background

Early administration of nutrition support is an important link in the chain of therapy for critically ill patients. Nasogastric and nasoenteral tube feeding plays an important role in the delivery of enteral nutrition (EN) in intensive care units (ICUs). Major guidelines [1–4] recommend postpyloric feeding in those critically ill patients at high risk of aspiration or with intolerance to gastric EN. The postpyloric feeding approach not only reduces gastrointestinal and respiratory complications in these patients but also ensures that the nutritional goals are better achieved [5–7]. Unfortunately, up to 79% of critically ill and surgical patients suffer from delayed gastric emptying [8]. A 7-year worldwide prevalence study of nutrition practice in the ICU indicated that only 5.5% of patients had a nasojejunal tube [9], partly attributable to a lack of effective transpyloric placement methods.

The optimal method of achieving safe and effective postpyloric enteral access at the bedside remains controversial [10]. Both endoscopic and fluoroscopic assistance are deemed the most effective methods for postpyloric tube placement [11, 12]. Several other methods, including electromagnetic, electrocardiographic, and ultrasonic guidance placement [13–15], are clinically available. However, these methods are highly device-dependent, and the success rates are variable [16, 17]. While the aforementioned resources are limited to access, using a self-propelled spiral tube for postpyloric feeding in critically ill patients is an alternative approach [18–22]. Nevertheless, the overall success rate of transpyloric migration via a spiral tube is relatively low despite using prokinetic agents [19, 20]. In the event that spontaneous transpyloric spiral tube migration used as a primary technique fails, a blind bedside method may be applied as a rescue technique. Various special techniques the blind bedside transpyloric tube placement have been introduced into clinical practice [23–27]. However, transpyloric spiral tube placement facilitated by a blind bedside method has not yet been reported. The objective of this prospective study was to evaluate the safety and efficiency of blind bedside spiral tube placement as a rescue therapy subsequent to failed spontaneous transpyloric migration in critically ill patients.

## Methods

### Study design

A prospective, multicenter, observational clinical trial was conducted in the ICUs of three tertiary hospitals. The study protocol met the Strengthening the Reporting of Observational Studies in Epidemiology (STROBE) criteria [28]. The ethics committee of Guangdong General Hospital and the other two participating hospitals approved the protocol (approval #GDREC2015425H(R1)). In accordance with the standards of the Declaration of Helsinki, a written informed consent form was obtained from each patient or from the next of kin for patients unable to consent. The trial was registered at http://www.chictr.org.cn (# ChiCTR-OPN-16008206) [29].

### Patients

Between April 2016 and February 2017, all patients who underwent spontaneous transpyloric spiral tube placement according to the eligibility and exclusion criteria described in a previous study [19], but who failed to gain spontaneous transpyloric migration despite using prokinetic agents, and still required EN for more than 3 days, were consecutively eligible for this study. Patients who were excluded from this study included: those with deterioration in medical conditions, such as uncontrolled shock, uncontrolled sepsis, uncontrolled gastrointestinal bleeding, emergency surgery, and so forth, or those transferred out of the ICU.

### Technique of blind bedside postpyloric placement of spiral tube

**Fig. 1:**
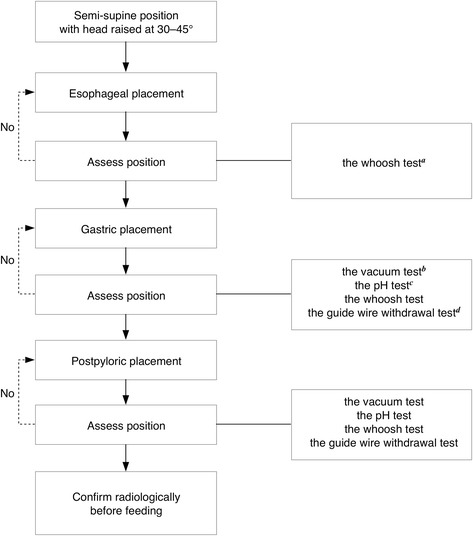
Method of blind bedside postpyloric spiral tube placement. ^a^The whoosh test was performed by air insufflation with auscultation in the epigastrium, in which a gurgling was regarded as indicative of air entering the stomach while the absence of gurgling suggested the tube tip was located elsewhere (lung, esophagus, pharynx, and so on). ^b^The vacuum test was done by instilling 60 ml air with a 20-ml syringe three times and then aspirated. If the volume of air aspirated was < 20 ml the tube was likely postpyloric, while if the volume of air aspirated was > 40 ml the tube was likely intragastric. ^c^The pH test was taken by measuring the pH value of aspiration with a pH strip. Aspiration of pH <5.0 was deemed intragastric. Aspiration of pH 6 − 7 was deemed to be from the small bowel. ^d^The guide wire withdrawal test was conducted by pulling back the guide wire a little way (within 5 cm). In a coiled tube, the guide wire either could be withdrawn with resistance felt as “popping”, or could be withdrawn easily but was unable to be re-inserted without a degree of force

Once failed spontaneous transpyloric migration was confirmed, this rescue method was applied on the same day (if confirmed on the day shift) or on the next morning (if confirmed on the night shift), to initiate timely postpyloric feeding without leveraging endoscopy or fluoroscopy. The technique of blind bedside postpyloric placement of a spiral tube was primarily described by Gatt et al. [23] and was routinely used in our hospital and the other two centers. Blind bedside postpyloric spiral tube placement was performed by a single intensivist in each center. A 145-cm-long spiral feeding tube made of radiopaque polyurethane (CH10, Flocare Bengmark, Nutricia, The Netherlands) was used. When failed spontaneous transpyloric migration was confirmed, the spiral tube was removed and sterilized for further use. The insertion technique involved three stages—esophageal, gastric, and postpyloric placement (Fig. 1). In contrast with the previous study [23], a spiral tube was used instead of a straight tube, and the tube was advanced approximately 100 cm rather than 115 cm. Another difference with previous methods of spiral tube placement is that the postpyloric placement was achieved by specific manipulation in minutes instead of spontaneous placement lasting hours or even days. Key to the success of this technique was the ascertaining of tube tip position at each stage before proceeding to the next.

#### Preparation and positioning

Patients were intravenously administered a bolus of 20 mg metoclopramide (or 10 mg in renal insufficiency) before placement of the tube. If there were no contraindications, the patient was placed semi-supine with the head of the bed raised at 30–45°. The distance from the ear, via the nasal tip, down to the xiphoid process was measured as an approximate estimate of the depth of insertion into the stomach. The feeding tube was then lubricated with paroline.

#### Esophageal placement

The tube was passed into the nostril, parallel to the nasal septum and hard palate, until the measured length was reached. At this level, esophageal placement was assessed by air insufflation with auscultation in the stomach (the whoosh test) [30]. If placement in the esophagus could not be verified, the tube was withdrawn back into the nose, and a further attempt was made. The crucial point was passing the tube through the epiglottis into the esophagus rather than through the glottis into the trachea.

#### Gastric placement

When the tube was advanced into the stomach, aspiration was attempted at 5-cm intervals till the gastric antrum was established. Aspiration with a 20-ml syringe was attempted three times at each level. The tube tip position was detected again using the whoosh test and the vacuum test by instilling and re-aspirating 60 ml of air [31]. The pH of any fluid aspirate was tested [32], and assessment for coiling was also performed using the guide wire withdrawal test [23].

#### Postpyloric placement

Postpyloric placement was accomplished by advancing the tube at 5-cm intervals and checking its position each time. In this manner the tube was advanced to about 100 cm. Once the 60 ml of insufflated air could only be aspirated with a minimal return, the tube was considered postpyloric; otherwise it was considered intragastric. Any fluid aspirate obtained was checked for pH, and the guide wire withdrawal test [23] was also used to assess the coiling of the tube in the stomach, which is the main difficulty encountered at this stage. Usually, the indwelling guide wire can be withdrawn easily in a coiled tube, but cannot be re-inserted without a degree of force. Notably, if withdrawing the guide wire required more force, the tube might have kinked considerably and needed to be pulled back to 55 cm for further attempt at pyloric intubation.

While the tube position was assessed to be postpyloric and the inserted length had reached about 100 cm, the required position was met. However, if the postpyloric position was achieved, for safety reasons we stopped further advancement when it required a degree of force. The tube was secured to the nose with adhesive tape, and the tube tip position was confirmed radiologically before feeding. In patients with obesity or gastrointestinal distension, which made the tube position difficult to review, an additional hydrosoluble contrast injection of meglumine diatrizoate was administered through the tube before radiography.

### Data collection

At baseline, the following data were assessed: demographic characteristics, diagnosis, concomitant medication, and severity of illness including the Acute Physiology and Chronic Health Evaluation II score, Sequential Organ Failure Assessment score, and Acute Gastrointestinal Injury grading.

During tube insertion, variables were documented, including time to insertion, length of insertion, number of attempts, and requirement of sedatives or analgesics. Adverse event data related to metoclopramide or insertion was also assessed and recorded. Vital signs including heart rate (HR), respiratory rate (RR), mean arterial pressure (MAP), and pulse oxygen saturation (SpO_2_) were monitored and transcribed every 5 min from the beginning to 30 min after the procedure. The radiologically confirmed tube tip position, which was reviewed by an expert group of intensivists and radiologists blinded to this study, was also recorded.

### Endpoints

The primary efficacy endpoint was the success rate of postpyloric spiral tube placement, defined as reaching the first portion of the duodenum or beyond. Secondary efficacy endpoints for blind bedside spiral tube placement included the success rate of spiral tube placement in D3 (the third portion of the duodenum) or beyond, rate of placement in the proximal jejunum, time to insertion, length of insertion, and number of attempts. Safety endpoints were metoclopramide-related adverse events and major adverse tube-associated events (MATEs), including vital sign alert events, requirement for sedatives or analgesics during the procedure, and so forth. A vital sign event was defined as HR, RR, or MAP fluctuating beyond the range of ± 15%, or pulse oxygen saturation declining to < 90%.

### Statistical analysis

Quantitative variables were presented as mean ± standard deviation or median (interquartile range) as appropriate, qualitative variables were presented as number (percentage). All statistical analyses were completed using SAS software (SAS v9.4; SAS Institute, NC, USA). A significance test was conducted using the paired rank-sum test for the difference when the data distribution was not normal or the paired Student's *t* test for the difference when the data distribution was normal. Statistical significance was set at the 5% level.

## Results

### Clinical and demographic data of patients

A total of 127 of 133 patients underwent postpyloric tube placement using the blind bedside technique from April 2016 to February 2017. Six patients were excluded due to ineligibility, of whom five patients were transferred out of the ICU and one patient progressed to uncontrolled sepsis. Tube placement was completed in all eligible patients, who were all included in the statistical analyses. Patients’ characteristics are presented in Table 1. Remarkably, of 127 patients, 66 received mechanical ventilation and 10 required vasopressor therapy.

**Table 1:** Clinical and demographic data of patients

Variables	Values in total study sample (n = 127)
Age, years	60 (48–72)
Gender (male)	86 (67.7)
Preexisting diseases	
Hypertension	27 (21.3)
Diabetic mellitus	9 (7.1)
Previous gastrointestinal surgery	4 (3.1)
Primary diagnosis	
Neurologic	53 (41.7)
Respiratory	36 (28.4)
Cardiovascular	15 (11.8)
Multiple trauma	14 (11.0)
Sepsis	5 (3.9)
Gastrointestinal	2 (1.6)
Others	2 (1.6)
Use of sedatives or analgesics	22 (11.0)
Use of vasopressors	10 (7.9)
Mechanical ventilation	66 (52.0)
APACHE II score	18 (13–23)
SOFA score	10 (8–12)
AGI grade	
Without AGI	5 (3.9)
I	11 (8.7)
II	92 (72.4)
III	19 (15.0)

Quantitative variables are presented as median (IQR) and qualitative variables as number (percentage)

*APACHE II* acute physiology and chronic health evaluation II, *SOFA* sequential organ failure assessment, *AGI* acute gastrointestinal injury, *SD* standard deviation, *IQR* interquartile range

### Primary efficacy endpoint and secondary efficacy endpoints

In 104 of 127 patients (81.9%, 95% confidence interval 75.2 − 88.2%), the feeding tubes were postpyloric, as summarized in Table 2. Of the 104 tubes positioned postpylorically, 70 (55.1%) were placed in D3 or beyond and 43 (33.9%) were placed in the proximal jejunum. Postpyloric placement was achieved in 61.4% of patients (78/127) at the first attempt, in 34.7% (44/127) at the second attempt and in 3.9% (5/127) at the third attempt. The placement procedure lasted a median time of 14 min, and the mean length of insertion was 95.6 cm.

**Table 2:** The primary endpoint and secondary efficacy endpoints

Endpoints	Value in total study sample (n = 127)
Primary endpoint	
Postpyloric placement^a^	104 (81.9)
Secondary endpoints	
Placed at D3^b^ or beyond	70 (55.1)
Placed at the proximal jejunum	43 (33.9)
Time to insertion, min	14 (10–15)
Number of attempts	1.4 ± 0.6
Length of insertion, cm	95.6 ± 9.3

Quantitative variables are presented as mean ± SD or median (IQR) as appropriate and qualitative variables as numbers (percentage)

^a^Postpyloric placement, reaching the first portion of the duodenum or beyond

^b^D3 is the third portion of the duodenum

In the patients in whom the procedure failed, endoscopy was used to achieve postpyloric placement in six patients on the next day. In the rest of the patients, the indwelling tubes served as a gastric feeding tube, because endoscopic or fluoroscopic assistance was limited in availability.

### Safety endpoints

The adverse event incidence was 26.0%, and no severe adverse event was observed (Table 3). Vital sign alert events, requirement of sedatives or analgesics during the procedure, and nausea were among the most frequent MATEs. The recorded metoclopramide-related adverse events were amyostasia, lethargy, dysphoria, and xerostomia. All these symptoms resolved within 24 h and did not require additional intervention.

**Table 3:** Adverse events

Events, number (percentage)	Value in total study sample (n = 127)
Any event	33 (26.0)
MATEs	29 (22.8)
Vital signs alert events^a^	15 (11.8)
Requirement of sedatives or analgesics during procedure	14 (11.2)
Nausea	8 (6.3)
Pain	6 (4.7)
Nasal mucosa bleeding	5 (3.9)
Vomiting	3 (2.4)
Metoclopramide-associated events	5 (4.0)
Amyostasia	2 (1.6)
Lethargy	1 (0.8)
Dysphoria	1 (0.8)
Xerostomia	1 (0.8)

Qualitative variables are presented as number (percentage)

*MATEs* major adverse tube-associated events

^a^Defined as any vital sign that fluctuated beyond the range of ± 15%, or pulse oxygen saturation that declined to < 90%

Vital signs were monitored during tube placement. It seemed that the HR, RR, and MAP increased slightly, while the SpO_2_ remained relatively stable. However, there was no statistical difference in vital signs assessed before and 30 min after tube placement (*P* > 0.05, Table 4).

**Table 4:** Vital signs monitored peri-procedure

Vital signs	Pre-procedure	Inter-procedure^a^	Post-procedure^b^	*P* value	
				Pre-procedure vs. Inter-procedure	Pre-procedure vs. Post-procedure
HR (bpm)	101.3 ± 25.2	105.6 ± 25.1	102.3 ± 25.2	<0.0001	0.1041
RR (rpm)	19.0 ± 5.5	20.5 ± 5.8	19.3 ± 5.3	<0.0001	0.1139
MAP (mmHg)	97.6 ± 14.0	99.9 ± 14.9	98.5 ± 14.4	<0.0001	0.1150
S_P_O_2_ (%)	98.6 ± 1.7	98.5 ± 1.6	98.6 ± 2.0	0.0789	0.9313

Quantitative variables are presented as mean (± SD)

*HR* heart rate, *RR* respiratory rate, *MAP* mean arterial pressure, *S*_*P*_ *O*_*2*_ pulse oxygen saturation, *bpm* beats per minute, *rpm* respirations per minute

^a^Inter-procedure, data were collected at the widest fluctuation point during the procedure

^b^Post-procedure, data were collected 30 min after the procedure

## Discussion

The present prospective cohort, using blind bedside spiral tube placement as a rescue therapy subsequent to failed spontaneous transpyloric migration in critically ill patients, demonstrated a success rate of 81.9% in achieving postpyloric access with a median time of 14 min. The adverse event incidence was 26.0%, and no serious adverse event was observed*.* Therefore, it is believed that this method of rescue therapy represents a significant improvement in applying postpyloric feeding, thereby obviating the more invasive fluoroscopic or endoscopic tube placement in a large number of patients. Therefore, it serves as a valuable method for transpyloric placement.

Several methods for transpyloric placement are available at present. Although fluoroscopic or endoscopic techniques are the most effective methods if the medical condition allows, these methods can be precluded due to radiation exposure, intra-hospital transportation risk, and poor timeliness. Recently, several techniques, including magnet-assisted placement, electrocardiographic guidance placement, and ultrasonic guidance placement, have been put into clinical practice, with an acceptable success rate illustrated in several relevant studies [13–15]. However, all these methods are device-dependent. Generally, these devices are expensive, and additional expenditure may be needed, thereby restricting their use in resource-limited settings. Spontaneous postpyloric placement [18–22] and several blind bedside methods [23–27] with varied success rates were developed to dispense from device dependence. In about 50% of subjects spontaneous self-advancing spiral tube placement fails to achieve postpyloric positioning despite use of prokinetics [19, 20]. In contrast, our “active” placement achieved post-pyloric placement in 81.9%. The success rate in achieving postpyloric access in the present study was similar or slightly lower, with a median time to insertion of 14 min, compared with previous studies on blind bedside methods [23–27]. The difference in success rate can be interpreted by a learning curve in a practitioner-dependent method [33, 34]. The success rate for jejunal access was 33.9% in the present study, which was significantly higher than that in a previous trial (10–19%) [19] but lower than that in the Cortrak studies (38.0–74.0%) [13, 35], or that reported by Gatt et al. (70.0%) [23]. This might be due to the heterogeneity among patients and different tubes. Another explanation could be the inconsistent length of insertion. Metheny et al. [5] found that aspiration pneumonia was significantly reduced when feeding tubes were placed in the small bowel, especially in the jejunum. It suggested that the rescue therapy may reduce the risk of aspiration and the incidence of aspiration-associated pneumonia. Therefore, it is valuable to validate in further studies whether increasing the length of insertion from the present 100 cm to 115 cm, consistent with that in Gatt et al. [23], can improve the success rate of jejunal access.

Adverse events were more frequent (26.0%) in the present study compared with other reports [23–27]. The most frequent MATEs were vital sign alert events, requirement of sedatives or analgesics during the procedure, and nausea. There are several possible explanations for this. The definition of safety endpoints in the present study, including vital sign alert episode and requirement for sedatives or analgesics during the procedure as adverse events, was quite different from that in other studies. Actually, vital sign alert event and requirement for sedatives or analgesics, which were not regarded as safety endpoints in other studies, constituted the predominant MATEs during the procedure. Moreover, metoclopramide-associated events were specifically observed and documented. Despite a transient fluctuation in vital signs, most cases returned to baseline within 30 min without additional intervention. Importantly, the majority of complications were minor, and no serious complication occurred. All these suggested that the current method represented a minimally invasive technique. Even though a low, even zero, complication rate was reported [23–27], severe complications [23, 36, 37] should be cautioned against due to the nature of the unguided nonvisual technique.

Although a similar or slightly lower success rate with an acceptable incidence of adverse events was presented in this cohort, while merging self-advancing tube placement as a primary technique with blind bedside postpyloric spiral tube placement as a rescue technique in cases of failed procedure into one, it is still an attractive resolution strategy. In fact, a relevant multicenter trial (ChiCTR-INR-16009099) [38] was registered and is ongoing. Accordingly, a novel resolution strategy with a promising success rate might evolve in the future.

This promising technique offers several advantages, and hence can be considered as a preferential method for postpyloric tube placement. First, this method avoids the training and cost required for device-dependent methods. Moreover, it can be performed at the bedside in a timely manner, which decreases the risks related to transportation. It is well-recognized that intra-hospital transportation is potentially hazardous or undesirable in critically ill settings [39, 40]. Furthermore, the procedure is substantially simplified compared with endoscopic or fluoroscopic placement. Therefore, this method might be easily reproducible by medical staff in other clinical care environments. It is believed that this technique should not be limited to specialized physicians.

The limitations of the present study included the study design that was observational and the fact that this was not a randomized controlled trial, especially lacking comparison with other methods. Another limitation might be that the impact of the learning curve on success rate was not taken into account, which is highly dependent on individual experience and ability.

## Conclusions

Blind bedside postpyloric placement of a spiral tube, as a rescue therapy subsequent to failed spontaneous transpyloric migration in critically ill patients, is safe and effective, and can be performed in a timely manner without additional equipment. This technique may facilitate early initiation of postpyloric feeding in the ICU.

## Abbreviations

AGI: Acute gastrointestinal injury
APACHE II: Acute Physiology and Chronic Health Evaluation II
CI: Confidence interval
D3: Third portion of the duodenum
EN: Enteral nutrition
HR: Heart rate
ICU: Intensive care unit
IQR: Interquartile range
MAP: Mean arterial pressure
MATE: Major adverse tube-associated event
*P*: *P* value
RR: Respiratory rate
SD: Standard deviation
SOFA: Sequential Organ Failure Assessment
SpO_2_: Pulse oxygen saturation
